# Editorial: Cell migration and tissue remodeling in infectious diseases

**DOI:** 10.3389/fcimb.2026.1848066

**Published:** 2026-04-28

**Authors:** Juliana P. B. de Menezes, Patricia S. T. Veras, Washington L. C. dos-Santos

**Affiliations:** 1 Laboratory of Host - Parasite Interaction and Epidemiology, Gonçalo Moniz Institute, Salvador, BA, Brazil; 2 Federal University of Bahia, Salvador, BA, Brazil

**Keywords:** cell adhesion, cell migration, host cells, immune response, pathogens

## Introduction

Pathogens are responsible for severe diseases that result in significant morbidity and mortality worldwide. During the pathogenesis of infectious diseases, cell migration and tissue remodeling play pivotal roles in shaping disease progression and clinical outcomes (Medzhitov, 2008; Wynn and Vannella, 2016). While these responses are essential for host defense, they may also contribute to tissue damage and environmental changes favoring pathogen persistence and coinfections (Nathan and Ding, 2010; Lei et al., 2025). Therefore, a deeper understanding of the mechanisms underlying host cell adhesion, migration, and tissue remodeling is critical for the development of innovative therapeutic strategies.

This Research Topic brings together cutting-edge studies that explore recent advances in our understanding of immune cell adhesion and migration, as well as tissue remodeling, in the context of infectious diseases. Collectively, these contributions provide important insights into the immunopathogenesis of infections and highlight potential avenues for the development of novel therapeutic approaches.

Zika virus (ZIKV) infection has been extensively associated with neurological and congenital abnormalities; however, its impact on other tissues, such as skeletal muscle, remains less well understood. In this Research Topic, the study entitled *“Zika virus infection disturbs development of human muscle progenitor cells”* provides important insights into how viral infection interferes with muscle regeneration processes. Using an *in vitro* model of human myogenesis, the authors demonstrate that ZIKV efficiently infects proliferating myoblasts, leading to alterations in key cellular functions. Infected cells exhibited impaired cell cycle progression, reduced proliferation, and significant defects in adhesion, migration, and membrane dynamics, all of which are essential for proper muscle development and repair. Moreover, ZIKV infection disrupted myoblast fusion, ultimately compromising the formation of mature myotubes. Interestingly, while differentiated myotubes were able to control viral replication, infected myoblasts displayed a markedly altered myogenic program. These findings highlight how ZIKV targets early stages of muscle cell differentiation, affecting fundamental processes such as cell migration and tissue remodeling, and suggest that viral interference with myogenesis may contribute to both developmental abnormalities and impaired muscle regeneration (Rocha et al.).

Expanding from virus-induced alterations in progenitor cell function to chronic infectious contexts, dysregulated cell migration and tissue remodeling are also central to the persistence of bacterial infections. In this context, the review entitled *“Wound Repair and Immune Function in the Pseudomonas infected CF Lung: Before and After Highly Effective Modulator Therapy”* discusses how impaired wound repair and immune responses shape the pathophysiology of cystic fibrosis (CF) lung disease. The authors highlight that persistent infection with *Pseudomonas aeruginosa* drives a cycle of chronic inflammation and defective tissue repair, in which epithelial damage, altered immune cell function, and unresolved injury perpetuate lung remodeling. Importantly, although highly effective cystic fibrosis transmembrane conductance regulator (CFTR) modulator therapies, such as elexacaftor/tezacaftor/ivacaftor, have significantly improved clinical outcomes and partially restored epithelial function, key processes involved in immune regulation and tissue repair do not fully return to a non-CF state. Moreover, incomplete eradication of *P. aeruginosa* and the potential for recurrent colonization underscore the continued disruption of host–pathogen interactions. This work emphasizes how persistent microbial presence and dysregulated immune cell dynamics sustain pathological tissue remodeling, reinforcing the need for complementary therapeutic strategies targeting inflammation, infection, and repair mechanisms (Matthews et al.).

At the molecular level, pathogen-driven modulation of host and parasite cellular machinery further illustrates the complexity of processes governing cell migration and tissue invasion. In this context, the study entitled *“EhVps35, a retromer component, is a key factor in secretion, motility, and tissue invasion by Entamoeba histolytica”* uncovers key mechanisms underlying parasite virulence. The authors demonstrate that EhVps35, a central component of the retromer complex, functionally interacts with the ESCRT machinery, forming a coordinated network involved in protein trafficking, secretion, and cellular remodeling. Notably, EhVps35 was shown to associate with a wide range of proteins and to be secreted in vesicles alongside ESCRT components, highlighting its role in vesicular transport pathways. Functional analyses revealed that silencing of EhVps35 significantly impairs trophozoite migration and reduces tissue invasion capacity, while also altering the localization of key proteins involved in membrane dynamics. These findings provide compelling evidence that intracellular trafficking and vesicle-mediated processes are critical regulators of parasite motility and host tissue invasion, reinforcing the concept that modulation of cellular architecture is a central strategy employed by pathogens to promote infection and dissemination (Diaz-Valdez et al.).

Further highlighting the molecular strategies employed by pathogens to modulate host–pathogen interactions, the study entitled *“A Toxoplasma gondii thioredoxin with cell adhesion and antioxidant function”* characterizes a multifunctional parasite protein involved in both redox balance and host cell interaction. The authors describe Trx21 as a thioredoxin-like protein containing glycosaminoglycan (GAG)-binding motifs and a transmembrane region, supporting its role in host cell adhesion. Functional analyses revealed that Trx21 exhibits antioxidant activity, protecting DNA from oxidative damage, while also mediating adhesion to host cells. These findings indicate that Trx21 contributes to parasite survival by integrating oxidative stress control with adhesion mechanisms, highlighting how *Toxoplasma gondii* exploits multifunctional proteins to facilitate host cell invasion, persistence, and modulation of the tissue microenvironment (Wang et al.).

Collectively, the studies included in this Research Topic provide a wide view of how infectious agents modulate cell migration and tissue remodeling across different biological contexts, ranging from alterations in progenitor cell function to chronic inflammation and finely tuned molecular mechanisms of pathogen virulence. These contributions highlight the multifaceted nature of host–pathogen interactions, in which both host responses and pathogen-derived factors converge to shape cellular dynamics and tissue architecture. Importantly, they also underscore that dysregulation of these processes is a central feature of disease pathogenesis, contributing to tissue damage, impaired repair, and pathogen persistence. Advancing our understanding of the mechanisms governing cell adhesion, migration, and remodeling in infectious diseases will be essential for the identification of novel therapeutic targets. Future studies integrating cellular, molecular, and translational approaches will be critical to develop innovative strategies aimed at restoring tissue homeostasis and improving clinical outcomes in infectious diseases.
